# *Plasmodium vivax* infection in Anajás, State of Pará: no differential resistance profile among Duffy-negative and Duffy-positive individuals

**DOI:** 10.1186/1475-2875-11-430

**Published:** 2012-12-22

**Authors:** Tarcisio AA Carvalho, Maíse G Queiroz, Greice L Cardoso, Isabela G Diniz, Aylla NLM Silva, Ana YN Pinto, João F Guerreiro

**Affiliations:** 1 Laboratório de Genética Humana e Médica, Instituto de Ciências Biológicas, Universidade Federal do Pará, Cidade Universitária Prof. José da Silva Neto, Rua Augusto Corrêa, N º 1, Guamá, CEP 66075-110, Belém, PA, Brasil; 2 Instituto Evandro Chagas, Programa de Malária, Belém, Pará, Brasil

**Keywords:** Vivax malaria, Duffy blood group, Brazilian Amazonia

## Abstract

**Background:**

There is large body of evidence that states that invasion of *Plasmodium vivax* requires the Duffy antigen, but the universality of this specificity is certainly now under question with recent reports showing that in some parts of the world *P*. *vivax* infects and causes disease in Duffy-negative people. These findings reinforce the idea that this parasite is rapidly evolving, being able to use other receptors than Duffy to invade the erythrocytes, which may have an enormous impact in *P*. *vivax* current distribution. The presence of *P*. *vivax* infection in Duffy-negative individuals was investigated in a cross-sectional study conducted in Anajás, Archipelago of Marajó, State of Pará, which is an area of malaria transmission in the Brazilian Amazonia.

**Methods:**

Duffy genotyping and *Plasmodium* species diagnostic assays were performed successfully in 678 individuals. An allele-specific primer polymerase chain reaction (PCR) technique was used for Duffy blood group genotyping. Identification of *Plasmodium* species was achieved by conventional blood smear light microscopy and a TaqMan-based real-time PCR method to detect mitochondrial genome of *Plasmodium falciparum* and *P*. *vivax*.

**Results:**

*Plasmodium spp*. infection was detected in 137 samples (20.2%). Prevalence of each *Plasmodium* species was 13.9% *P*. *vivax*, 5.8% *P*. *falciparum*, and 0.6% *P*. *vivax* plus *P*. *falciparum*. Overall, 4.3% (29/678) were genotyped as Duffy-negative (FY*B^*ES*^/*B^*ES*^). Among Duffy-negative individuals 6.9% were *P*. *vivax* PCR positive and among Duffy-positive 14.2% were *P*. *vivax* PCR positive. Although lower, the risk of Duffy-negatives to experience a *P*. *vivax* blood stage infection was not significantly different to that of Duffy-positives. Furthermore, the genotypic and allelic frequencies of the Duffy blood group among *P*. *vivax*-infected patients and in the control group did not differ significantly, also suggesting no reduction in infection rates among the carriers of FY*B^*ES*^ allele.

**Conclusions:**

The data obtained in Anajás showed no differential resistance vivax malaria among Duffy-negative and Duffy-positive individuals. This result needs additional confirmation through a deeper evaluation in a larger sample of patients with *P*. *vivax* malaria and molecular parasite characterization. Nonetheless, this genetic profile of the parasite may be contributing to the high incidence of malaria in the municipality.

## Background

Five species, *Plasmodium malariae*, *Plasmodium ovale*, *Plasmodium falciparum*, *Plasmodium vivax* and, recently, *Plasmodium knowlesi*, are recognized as natural malaria parasites of humans. Due to its biology, *P*. *vivax* is the most widely transmitted, occurring in temperate latitudes, arid regions, at high altitude and in other environments that are inhospitable to *P*. *falciparum*. Outside tropical Africa, in Asia and the Western Pacific, and in Central and South America, the most prevalent of these parasites is *P*. *vivax*. By contrast, in sub-Saharan Africa, where malaria transmission is otherwise more intense than anywhere else in the world, cases of *P*. *vivax* are greatly outnumbered by those due to *P*. *falciparum*, and *P*. *vivax* is almost undetectable in the local human populations. In Brazil, the incidence of malaria is almost exclusively (99.8%) of the cases restricted to the region of the Amazon Basin, and is caused by three species of *Plasmodium*: *P*. *vivax* (that accounts for 83.7% of the registered cases), *P*. *falciparum* (causing 16.3% of the cases) and *P*. *malariae* (rarely observed). No autochthonous transmission of *P*. *ovale* and *P*. *knowlesi* occurs [1,2].

The gap in distribution of *P*. *vivax* in Africa compared to the rest of the world is viewed as the consequence of the lack of expression of the Duffy antigen on the red cells (Duffy-negative phenotype), which mediates invasion of reticulocytes by *P*. *vivax*, and is highly predominant in the African populations in contrary to the observed in European, Asian and American populations in whom the Duffy-positive phenotype is more common. The pattern of Duffy-blood group distribution is attributed to a positive natural selection, since *P*. *vivax* requires the presence of Duffy antigen receptor for chemokines on the red blood cells’ (RBC) surface to be able to invade cells and cause disease (reviewed in [3-6]).

The Duffy blood group locus, at position q21–q25 on chromosome 1 [7], is characterized by three main alleles: FY*A, FY*B and FY* *B*^*ES*^. The FY*A and FY*B alleles are distinguished by a missense mutation, which results in a single amino acid difference and gives the common Fy (a+b-), Fy(a-b+) and Fy(a+b+) phenotypes [8-11]. The FY*B^*ES*^ allele, which corresponds to the Fy(a-b-) serological phenotype (i e, the absence of Fy antigen), is due to a T-33C point mutation on the FY*B gene promoter, which abolishes the erythroid gene expression by disrupting a binding site for the GATA-1 erythroid transcription factor and results in the elimination of the transcription of FY mRNA in RBCs, but not in other cell types [12,13]. The same mutation associated with the FY*A variant (FY*A^*ES*^ allele) was already identified at low frequencies in individuals living in a *P*. *vivax*-endemic region of Papua New Guinea [14,15].

Although there is a large body of evidence that states that invasion of *P*. *vivax* requires the Duffy antigen, the universality of the specificity is certainly now under question with recent reports that in some parts of the world *P*. *vivax* infects and causes disease in Duffy-negative people: in western Kenya [16], in the Western Brazilian Amazon region [17,18], in Madagascar [19], in African West Coast (Equatorial Guinea and Angola) [20], and in Mauritania, north-west Africa [21]. These findings reinforce the idea that this parasite is rapidly evolving, being able to use other receptors than Duffy to invade the erythrocytes, which may have an enormous impact in *P*. *vivax* current distribution [20].

The aims of this study were to investigate the presence of *P*. *vivax* infection in Duffy-negative individuals from Anajás, State of Pará, an area of malaria transmission in the Brazilian Amazonia, using an allele-specific primer polymerase chain reaction (PCR) technique for Duffy blood group genotyping, and conventional blood smear light microscopy and a TaqMan-based real-time PCR method to detect *P*. *falciparum* and *P*. *vivax*.

## Methods

### Ethics statement

The study was approved by the Ethics Committee of João de Barros Barreto Hospital, Federal University of Pará, Belém, State of Pará, Brazil, and health authorities from the municipal district of Anajás.

### Sampling

The samples for this study were collected in August 2009 in a cross-sectional study conducted in Anajás, Archipelago of Marajó, State of Pará, Eastern Brazilian Amazon (00°59'13''S; 49°56'24''W). Anajás was the municipal district that presented the highest index of malaria in the State of Pará in the year of 2009, with 26,043 positive cases in a population of 27,385 inhabitants, resulting in an Annual Parasitic Index (IPA) of 951/1,000 inhabitants [22].

In total, 738 patients were examined, 339 of them living in the city and 399 in two riverine communities in rural Anajás (Vencedora, n=212 and Luciana, n=187). Luciana village is located on the left bank of the River Mocoões (0°50'27.81''S;49°50'9.14"W), about 25 km downstream of the city of Anajás. Vencedora village is located on the right bank of the Alto Rio Anajás (0°58'52.26''S;49°58'08.33"W), about 5 km upstream from the city of Anajás. The patients were clinically examined, and 2 mL of blood were drawn for thick blood smears preparation and for molecular diagnosis of malaria infection. Microscopic parasitaemia examinations were performed by three experienced malaria field microscopists from the Federal University of Pará (UFPA) on slides using the thick film method and the results were reported as parasites/μL [23].

### DNA extraction

DNA was extracted from 300 μL of EDTA-treated blood using the NeoIsoColumn kit (One Lambda Inc., San Diego, CA, USA) according to the manufacturer’s instructions. DNA was eluted in 200 μL of elution buffer (provided with the kit).

### Real-time PCR

For real-time PCR, Primer Express software (Life Technologies, Foster City, CA, USA) was used to design specific primers (forward and reverse) targeting a mitochondrial DNA (mtDNA) sequence common to all *Plasmodium spp*, and TaqMan™ fluorescence-labelled probes to hybridize differentially in *P*. *vivax* or *P*. *falciparum*, enabling species identification. The following oligonucleotides primers and probes were used: forward 5’-ACCTCCAGGCAAAGAAAATGAC-3’, reverse 5’-GGCGAGAAGGGAAGTGTGTTT-3’ and probes 5’-AACGGAATCAGTTAA-3’-FAM for *P*. *vivax* and 5’-ACGGAATCAATTAAC-3’-VIC for *P*. *falciparum*. DNA templates were amplified in an Applied Bio-systems 7500 analytical PCR system (SDS version 1.7). Briefly, a 50 μL PCR mixture was performed using 20–100 ng/μL of purified DNA template, 25 mL of TaqMan™ 2X (Life Technologies) universal PCR master mix, and a final concentration of 300 nM of each parasite species-specific primer and 200 nM of each corresponding probe. Amplification and detection were performed under the following conditions: 2 min at 50°C to achieve optimal AmpErase uracil-N-glycosylase activity, 10 min at 95°C to activate the AmpliTaq Gold DNA polymerase, and 45 cycles of 15 sec at 95°C and 1 min at 60°C using a 7500 Real-Time PCR System (Life Technologies, Foster City, CA, USA). Each experiment included one reaction mixture without DNA as a negative control.

### DNA sequence analysis

The specificity of the assay was confirmed by sequencing the PCR products from all positive samples using a Big Dye terminator sequencing kit (Applied Biosystems) on an ABI 3130 sequencer (Applied Biosystems) following the manufacturer’s instructions. In samples with positive results to *P*. *falciparum*, this procedure is particularly necessary to exclude possible infections by *P*. *malariae*, *P*. *knowlesi* and *P*. *ovale*, since the employed probe can identify all these species. The sequences obtained were aligned with those deposited in GenBank using the BLAST (Basic Local Alignment Search Tool) program.

### Duffy blood group genotyping

The samples were genotyped using an allele-specific primer polymerase chain reaction (PCR) technique described by Olsson *et al*[24]. Amplification was performed for each subject with sense primers corresponding to normal and GATA-1-mutated promoter sequence combined with antisense primers that discriminate the FY*A and FY*B alleles in four different combinations of primer pairs. PCR mixtures included 100 ng genomic DNA, 0.2 mM of each primer, 100 mM of each dNTP, 1.5 mM MgCl2, and 0.5 U AmpliTaq Gold polymerase (Perkin Elmer, USA) in the AmpliTaq Gold buffer supplied by Perkin Elmer in a reaction volume of 25 μL. Mixtures were incubated for 8 min at 95°C, followed by 10 cycles of 94°C for 1 min and 69°C for 1 min, 25 cycles of 94°C for 1 min, 64°C for 1 min and 72°C for 1 min, and a final incubation at 72°C for 10 min. PCR products were separated electrophoretically using 1.5% agarose gel at 150 volts for 30 min and visualized with SYBR® Safe DNA gel stain under UV excitation.

### Statistical analysis

Statistical analysis was undertaken using χ^2^ analysis to compare the proportions of Duffy genotypes alleles among in *P*. *vivax*-infected patients and sympatric malaria-exposed controls (*P*. *falciparum*-infected and non-infected individuals).

## Results

Both Duffy genotyping and *Plasmodium* species diagnostic assays (qPCR-mtDNA method) were performed successfully for 678 individuals (330 urban, 166 of Vencedora and 182 of Luciana). *Plasmodium spp*. infection was detected in 137 samples (20.2%), of which 57 (17.3%) urban, 25 (15.1%) of Vencedora and 55 (30.2%) of Luciana. Overall prevalence of each *Plasmodium* species was 13.9% (94/678) *P*. *vivax*, 5.8% (39/678) *P*. *falciparum*, and 0.6% (4/678) *P*. *vivax* + *P*. *falciparum*. In all of the study sites prevalence of *P*. *vivax* was the highest, ranging from 12.1% in urban patients to 17.6% in Villa Luciana. P. falciparum was found in only one patient of Vencedora (0.6%), 16 (4.8%) patients and in 22 urban (12.1%) of Luciana. Overall, 4.3% (29/678) were genotyped as Duffy-negative (FY*BES/*BES) FY*B^*ES*^/*B^*ES*^ and 95.7% (649/678) were Duffy positive. Among Duffy-negative individuals 6.9% (2/29) were *P*. *vivax* PCR positive based on the qPCR-mtDNA method, and among Duffy-positive 14.7% (96/649) were *P*. *vivax* PCR positive. The two Duffy-negative PCR positive for *P*. *vivax* were identified at Luciana (2/22 infected individuals). The risk of Duffy-negatives to experience a *P*. *vivax* blood stage infection was lower but not significantly different to that of Duffy-positives (Odds ratio = 0.4460; 95% confidence interval 0.1044 – 1.9060; P = 0.3983).

Moreover, among *P*. *vivax*-infected patients (94 *P*. *vivax* mono-infections and four *P*. *vivax*/*P*. *falciparum* mixed infections), 2.0% were Duffy-negative and the frequency of the allele FY*B^*ES*^ in this group was 18.6%. Among P. *falciparum*-infected and non-infected individuals (sympatric malaria-exposed controls, n = 584) 4.6% were Duffy-negative (FY*B^*ES*^ allele frequency of 18.3%). In the total sample the frequency of allele FY*B^*ES*^ was 18.4%. No significant differences were observed when comparing the genotypic and allelic frequencies of the Duffy blood group among Duffy-negative *P*. *vivax*-infected patients and controls both the total sample and in each of the three study sites, also suggesting no significant reduction of infection rates among the carriers of the FY*B^*ES*^ allele (Tables 1 and 2).

**Table 1:** **Duffy genotyping and *Plasmodium *species diagnosis in the population of Anajás**, **State of Pará**, **Brazil**

**Place**	**Duffy genotypes**	Controls*	***P***. ***vivax***-**infecteds**	**p**-**value**
City of Anajás	FY*A/*A	83 (28.6%)	12 (30.0%)	0.9955
	FY*A/*B	97 (33.4%)	10 (25.0%)	0.3735
	FY*A/*BES	40 (13.8%)	7 (17.5%)	0.6983
	FY*B/*B	36 (12.4%)	7 (17.5%)	0.5188
	FY*B/*BES	24 (8.3%)	4 (10.0%)	0.9488
	FY*BES/*BES	10 (3.4%)	0 (0.0%)	0.4835
Vencedora	FY*A/*A	29 (20.1%)	5 (22.7%)	0.9973
	FY*A/*B	44 (30.6%)	4 (18.2%)	0.3473
	FY*A/*B^*ES*^	21 (14.6%)	3 (13.6%)	0.8354
	FY*B/*B	19 (13.2%)	4 (18.2%)	0.7647
	FY*B/*B^*ES*^	26 (18.1%)	4 (18.2%)	0.7771
	FY*B^*ES*^/*B^*ES*^	5 (3.5%)	2 (9.1%)	0.5145
Luciana	FY*A/*A	36 (24.0%)	8 (25.0%)	0.9144
	FY*A/*B	29 (19.3%)	7 (21.9%)	0.9336
	FY*A/*B^*ES*^	29 (19.3%)	5 (15.6%)	0.8112
	FY*B/*B	24 (16.0%)	4 (12.5%)	0.8194
	FY*B/*B^*ES*^	20 (13.3%)	8 (25.0%)	0.1643
	FY*B^*ES*^/*B^*ES*^	12 (8.0%)	0 (0.0%)	0.2065
Total population	FY*A/*A	147 (25.3%)	26 (26.5%)	0.2106
	FY*A/*B	169 (29.1%)	22 (22.4%)	0.8606
	FY*A/*BES	90 (15.5%)	15 (15.3%)	0.6765
	FY*B/*B	78 (13.4%)	16 (16.3%)	0.9430
	FY*B/*B^*ES*^	69 (11.9%)	17 (17.3%)	0.0662
	FY*B^*ES*^/*B^*ES*^	27 (4.7%)	2 (2.0%)	0.2634

*patients infected with *P*. *falciparum* and non-infected individuals.

**Table 2:** **Allelic frequencies of the Duffy blood group system and *Plasmodium *species diagnosis in in the population of Anajás**, **State of Pará**, **Brazil**

**Place**	**Alelles**	Controls*	**P**. ***vivax***-**infecteds**	**p**-**value**
City of Anajás	FY*A	303 (52.2%)	41 (51.3%)	0.9625
	FY*B	193 (33.3%)	28 (35.0%)	0.8572
	FY*B^*ES*^	84 (14.5%)	11 (13.8%)	0.9959
Vencedora	FY*A	123 (42.7%)	17 (38.6%)	0.7297
	FY*B	108 (37.5%)	16 (36.4%)	0.9823
	FY*B^*ES*^	57 (19.8%)	11 (25.0%)	0.5507
Luciana	FY*A	130 (43.3%)	28 (43.8%)	0.9380
	FY*B	97 (32.3%)	23 (35.9%)	0.6815
	FY*B^*ES*^	73 (24.3%)	13 (20.3%)	0.5993
Total population	FY*A	553 (47.7)	89 (45.4)	0.5378
	FY*B	394 (34.0)	71 (36.2)	0.5571
	FY*B^*ES*^	213 (18.4)	36 (18.4)	0.9986

*patients infected with *P*. *falciparum* and non-infected individuals.

## Discussion

The prevalence of *P*. *vivax* and *P*. *falciparum* and Duffy-blood group genotype distribution was studied in the population of Anajás, State of Pará, an area of malaria transmission in the Brazilian Amazonian, in order to analyse the presence of *P*. *vivax* infection in Duffy-negative individuals.

Until recently, the Duffy-negative phenotype was seen as giving complete protection against infection by *P*. *vivax*, since this parasite requires the presence of Duffy antigen receptor for chemokines on the RBC surface to be able to invade cells and cause disease. However, the universality of this specificity has been questioned by recent reports that *P*. *vivax* infects and causes disease in Duffy-negative people in some parts of the world [16-21].

In this study, 6.9% (2/29) of the Duffy-negative subjects were diagnosed as *P*. *vivax*-infected, a finding that confirms previous reports in patients from Rondonia, western Brazilian Amazon [17,18], as well as in Kenya, East Africa [16], in Madagascar [19], Equatorial Guinea and Angola, African West Coast [20] and Mauritania, north-western Africa [21]. Moreover, the data obtained in the population of Anajás are in accordance with those found in children from Madagascar [19], where in individual study sites with sufficient numbers of PCR-positive *P*. *vivax* infections to enable comparisons, prevalence ratios were not significantly different between Duffy-positive and -negative children. That is, the risk of malaria infection due to *P*. *vivax* was not different between the Duffy-negative and Duffy-positive groups.

It is of note that other genotypes besides Duffy-negative have been shown capable of influencing the susceptibility to *P*. *vivax* infection [18,25], but this was not observed in Anajás, since the genotype frequencies did not differ significantly between controls and *P*. *vivax*-infected patients both in the whole sample and in each site investigated. One possible explanation for the results obtained in Anajás would be that the intensity of transmission of *P*. *vivax* in this region is such that it provides a constant source of parasites that infect Duffy-positives, providing ample opportunities for infection of hepatocytes Duffy-negative and selecting strains of *P*. *vivax* with a new capacity to invade erythrocytes, possibly through a Duffy-independent mechanism. In this scenario, less prominent protective effects against infection by *P*. *vivax* conferred by other genotypes seem to have been abolished or are less evident.

This study was unable to assess possible effects of Duffy genotypes on the risk of developing clinical malaria, since the patients were not followed after medical attention. Anyway, it is important to note that only one of the two Duffy-negative patients diagnosed as *P*. *vivax*-infected through mtDNA-qPCR method was confirmed by microscopic examination of blood smear, and had parasitemia classified as low (12 parasites per field under oil immersion microscope). The other patient was only detected by qPCR, but also presented a profile consistent with low parasitemia. In addition, with respect to clinical manifestations, both were asymptomatic at the time of medical consultation.

The observed frequencies of Duffy-negative genotype in *P*. *vivax* patients and controls in Anajás population (2.1% and 4.6%, respectively), which are not significantly different, are smaller than those found in Afro-Brazilian communities in eastern Amazonia (Pará and Amapá) [26], in which the genotype frequencies ranged from 0.323 to 0.588, but are quite similar to those found in most Amazonian populations already studied (frequencies ranging from 0 to 12%) [17,18,27,28]. Moreover, the frequencies of Duffy genotypes found in Anajás are the expected for a population with a genetic background resulting from the admixture between Europeans, mainly Portuguese, Africans and Amerindians in very close proportions [29]. Thus, the observed distribution of Duffy genotypes in the population of Anajás with a high frequency of Duffy-positive associated with a high prevalence of malaria, predominantly *P*. *vivax*, appears to fulfil the conditions considered by Ménard *et al*[19] as necessary to clear the barrier of Duffy negativity, providing conditions for the parasites have sufficient exposure to Duffy-negative red cells, allowing more opportunities for *de novo* selection or optimization of an otherwise cryptic invasion pathway that nevertheless seems less efficient than the Duffy-dependent pathway.

## Conclusions

The data obtained in the population of Anajás showed no differential resistance to *P*. *vivax* infection among Duffy-negative and Duffy-positive, a result that needs to be corroborated by further evaluation in a larger sample of patients with *P*. *vivax* malaria, and by molecular characterization of the parasite in order to evaluate the diversity of strains of *P*. *vivax* circulating in this area, coupled with the ability to invade erythrocytes using other receptors than Duffy. However, this result could mean that new capacity of the parasite may be relatively common in the population of Anajás through adaptive mechanisms and evolutionary processes that deserve to be investigated, and that this genetic profile of the parasite, resulting in the loss of an important protective mechanism against vivax malaria, may be contributing significantly to increase the susceptibility to infection by *P*. *vivax* and, consequently, to the high incidence of malaria in the municipality of Anajás.

## Competing interests

The authors declare that they have no competing interests.

## Authors’ contributions

JFG, TAAC conceived and designed the experiments; TAAC, MGQ, GLC, IGD performed the experiments; JFG analysed the data wrote the paper; JFG, TAAC, MGQ, AYNP carried out the biological material and data collection in the field. All authors read and approved the final manuscript.
